# National Emergency Department Registry for Mpox: Coordinating Multisite Surveillance Networks to Inform Current and Future Outbreaks

**DOI:** 10.1016/j.acepjo.2025.100212

**Published:** 2025-06-28

**Authors:** Gaby Dashler, Kiran Faryar, Kimberly A. Stanford, Jonathan W. Dyal, Robert C. Doerning, Elissa M. Schechter-Perkins, Jason W. Wilson, Larissa S. May, Herbert C. Duber, Yu-Hsiang Hsieh, Richard E. Rothman, Bhakti Hansoti

**Affiliations:** 1 Department of Emergency Medicine, Johns Hopkins University School of Medicine, Baltimore, Maryland, USA; 2 Department of Emergency Medicine, University Hospitals Cleveland Medical Center, Cleveland, Ohio, USA; 3 Department of Medicine, Section of Emergency Medicine, University of Chicago, Chicago, Illinois, USA; 4 Department of Emergency Medicine, University of Texas Health Science Center at Houston, Houston, Texas, USA; 5 Department of Emergency Medicine, University of Washington, Seattle, Washington, USA; 6 Department of Emergency Medicine, Boston Medical Center/Boston University Chobanian and Avedisian School of Medicine, Boston, Massachusetts, USA; 7 Emergency Medicine, University of South Florida Health Morsani College of Medicine, Tampa, Florida, USA; 8 Department of Emergency Medicine, University of California Davis Health, Sacramento, California, USA

**Keywords:** Mpox, emergency department, surveillance, infectious disease, Public Health Surveillance, emerging infections, sexually transmitted infections

## Abstract

**Objectives:**

Despite many emergency departments (EDs) managing patients with possible mpox, individual institution reports have been limited, making it challenging to understand demographic trends and service needs. We established a data repository of ED patients with mpox to inform epidemiologic trends and future practice.

**Methods:**

This national multicenter retrospective chart review included patients aged ≥ 18 years presenting to 8 EDs across the United States during the mpox outbreak (June 2022 to January 2023) with laboratory-confirmed mpox. Data were collected on demographic variables and clinical characteristics from each participating site, representing the northeast, midwest, south, and west regions.

**Results:**

A total of 270 patients were included in the cohort; most were men (264, 98%) and relatively young (median age: 34 years; IQR: 28-41). Patients commonly presented with skin lesions (253, 94%), fever or flu-like symptoms (152, 56%), and rectal pain (70, 26%). More than half (141/270, 52%) of individuals had a known diagnosis of HIV. Among those tested for sexually transmitted infection (STI) coinfection, 36% (51/143) had positive syphilis serology (not all diagnoses could be confirmed by clinical history), followed by gonorrhea 7% (9/128) and chlamydia 7% (8/122).

**Conclusion:**

Similar to the COVID-19 pandemic, EDs played a critical role in the mpox response. High coinfection rates with STIs highlight the need for routine coinfection testing. Multisite data collection identified key trends, emphasizing the value of surveillance networks for outbreak response.

**The Bottom Line**

This study of 270 mpox cases from 8 US emergency departments found high rates of coinfection with HIV (52%) and syphilis (36%), significantly exceeding national estimates. Most patients were young men, with 20% requiring admission. Notably, 15% of mpox patients were newly diagnosed with HIV in the emergency department, highlighting the need for routine HIV testing in suspected mpox cases. Regional differences were observed, with higher MSM contact in the northeast and more uninsured cases in the south. Findings underscore the importance of multisite surveillance to inform targeted public health responses.

## 1 Introduction

### 1.1 Background

On July 23, 2022, the World Health Organization (WHO) declared the mpox outbreak a public health emergency of international concern.^1^ Since 2022, over 90,000 cases have been documented across 118 countries, with 94% occurring in regions that had not historically reported mpox.^2^ Most literature on the 2022 mpox outbreak originates from outside the United States, primarily from outpatient settings like sexual health and primary care clinics.^3^, ^4^, ^5^, ^6^, ^7^, ^8^, ^9^, ^10^, ^11^ In contrast, emergency medicine publications have mainly focused on clinical management rather than epidemiologic data.^12^, ^13^, ^14^ Despite low hospitalization and case fatality rates, the ED serves as a critical surveillance site, particularly for vulnerable patients with limited healthcare access.^15^^,^^16^

Many EDs have evaluated and managed patients with suspected mpox, but individual site reports have been relatively small, limiting the understanding of demographic trends and service delivery needs.^3^^,^^5^^,^^7^, ^8^, ^9^, ^10^, ^11^ Given the ED’s role as a frontline and safety net site, understanding the epidemiology and management of mpox in this setting is essential.^17^

### 1.2 Objective

The Emergency Medicine Transmissible Infectious Diseases and Epidemics (EMTIDE) Consortium is a national network of emergency physicians, researchers, and public health stakeholders. In 2023, EMTIDE developed a multi-institutional data sharing platform to create a pooled repository of mpox cases in EDs, aiming to inform clinical practice and health policy. Although EMTIDE operates independently, its data collection complements efforts by public health agencies like the Centers for Disease Control and Prevention (CDC) and WHO. Unlike broader surveillance data, which often dilute ED-specific cases when integrated with other health care settings, EMTIDE provides a focused analysis of ED-based epidemiology, offering a complementary perspective to existing public health reports.

### 1.3 Goals of This Investigation

This study represents an epidemiologic analysis of mpox cases among ED patients across a national network, enhancing the understanding of mpox presentation and management within the ED setting.

## 2 Methods

### 2.1 Study Design and Setting

This is a national multicenter retrospective chart review of patients presenting to 8 geographically disparate EDs during the mpox outbreak from June 2022 to January 2023 with laboratory-confirmed mpox. All study site EDs were affiliated with an academic institution and had the ability to confirm mpox diagnosis on site. Participating sites included patients who presented to the institution’s academic tertiary care ED as well as their affiliated community sites. Johns Hopkins University served as the data coordinating center.

### 2.2 EMTIDE Consortium and Study Sites

This study was conducted as a part of the EMTIDE consortium, which consists of emergency medicine researchers who voluntarily contribute to select research projects at the intersection of emergency medicine and infectious diseases. All participants of the EMTIDE consortium were notified of this study and invited to participate during regularly scheduled monthly meetings and via email. Emergency medicine researchers within the EMTIDE network voluntarily opted to participate based on their regional mpox infection burden and data availability.

All sites had the ability to identify patients at their site who had laboratory-confirmed mpox and were willing to submit local IRB approval for retrospective chart review and data sharing with a national registry. A total of 8 sites participated in the study namely; Johns Hopkins and Boston Medical Center in the northeast, University Hospitals Cleveland Medical Center and University of Chicago in the midwest, the University of South Florida and University of Texas in the south, and University of California (UC) Davis and University of Washington in the west.

### 2.3 Selection of Participants

Eligible patients included ED patients aged ≥ 18 years old who had a laboratory-confirmed diagnosis of mpox.

### 2.4 Data Collection

The primary data source for this study was the electronic medical record, used to identify laboratory-confirmed cases during the study period. Each site contributed a limited data set (dates but no patient identifiers), manually abstracted by trained local staff who were blinded to the study objectives. Data were directly entered into a REDCap database at Johns Hopkins University. Patient identifiers (medical record number [MRN], name, and date of birth) were used locally to identify cases but were not shared on the master database.

Data variables were collected using a standardized form. Missing data, resulting from documentation gaps, were recorded as “unknown” to preserve the full sample. Missingness is clearly reported to assess its potential impact.

Demographic variables included age, race, ethnicity, sex at birth, insurance status, and ED encounter date. Age was recorded as a continuous variable (median and IQR reported). Race was categorized as Black or African American, White, other, or unknown, and ethnicity as Hispanic/Latino, non-Hispanic/Latino, other, or unknown. Sex at birth was classified as male or female, and insurance status as private, Medicaid, Medicare, uninsured, or unknown.

Clinical characteristics included presenting symptoms, emergency severity index (ESI), disposition, and follow-up. Symptoms were categorized as skin lesions, oral lesions, rectal lesions, fever/flu-like symptoms, and rectal pain. ESI levels were classified from level 1 (resuscitation) to level 5 (nonurgent). Suspected transmission routes were recorded as men who have sex with men (MSM), multiple partners, regular partner or spouse, casual partner, nonsexual contact, or unknown. Disposition was categorized as discharged or admitted, and follow-up as attended, not attended, or unknown.

Coinfection data included HIV status, other STIs, and microbiology results. HIV status was recorded as positive, negative, or unknown. Other STIs were documented based on laboratory results, with each pathogen recorded as positive, negative, or not performed.

### 2.5 Outcomes and Analysis

The primary outcome was the number of patients presenting to the ED with mpox. Secondary outcomes included the number and proportion of patients who underwent STI cotesting and had identified coinfections (HIV, chlamydia, gonorrhea, and syphilis), as well as other comorbidities and ED disposition. Geographic regions were classified using the US Census Bureau definition. Descriptive statistics were performed using Stata V18.0 (StataCorp) to summarize demographics, clinical presentations, and coinfection rates. Age differences across regions were assessed using the Shapiro-Wilk test for normality. Due to non-normal distributions in the midwest and south (*P* < .05), the Kruskal-Wallis test was used to compare median ages, reported as median and IQR. For categorical variables, the χ^2^ test of independence was used, and adjusted residuals were examined when *P* < .05. To identify factors associated with ED admission, we performed multivariable logistic regression, including demographics, clinical symptoms, emergency severity index (ESI), and HIV status. Significant predictors (*P* < .05) were reported as odds ratios (OR) with 95% CI. Model diagnostics addressed multicollinearity and perfect prediction using penalized regression or combining sparse categories as needed.

### 2.6 IRB Approval and Consent

All sites received Institutional Review Board (IRB) approval for retrospective chart review across the various contributing sites; a waiver of informed consent was sought. Johns Hopkins University received approval to create a research registry and store a limited data set using a research-compliant institutional REDCap platform. A data use agreement was executed between Johns Hopkins University and collaborating sites to directly upload a limited data set to the REDCap database and share data across sites that contributed to the data set.

## 3 Results

### 3.1 Demographic Characteristics

A total of 270 patients were diagnosed with mpox across 8 study sites from June 2022 to January 2023. Most patients were men (98%), with a median age of 34 years (IQR: 28-41; Table 1). Significant differences were observed across regions for race, ethnicity, and insurance status. The χ^2^ test revealed a significant difference in race distribution (χ^2^(9) = 78.19, *P* < .001). Sites in the northeast (53%) and midwest (89%) had a higher proportion of Black or African American patients, whereas the west (63%) had a higher proportion of White patients. In the south, 37% of patients reported “Other” as their race. Ethnicity distribution also varied significantly (χ^2^(9) = 49.92, *P* < .001), with 30% of patients in the south identifying as Hispanic or Latino, compared with predominantly non-Hispanic populations in other regions. Insurance status differences were also significant (χ^2^(12) = 100.52, *P* < .001). The south had the highest proportion of uninsured patients (53%), whereas the midwest and west had higher proportions of Medicaid clients (60%). In the northeast, 33% of the patients had private insurance, whereas 27% had Medicaid. Similarly, in the west, 33% had private insurance, but with a greater proportion of Medicaid coverage compared with the northeast.

**Table 1:** Demographic and clinical characteristics by region.

	Total (N = 98)	Northeast (N = 58)	Midwest (N = 44)	South (N = 120)	West (N = 48)	*P* value
Age, median (IQR)	34 (28-41)	35 (28-40)	31 (26-39)	34 (29-42)	35 (28-43)	
Race/Ethnicity						<.001∗
Black or African American	125 (46.3)	31 (53.5)	39 (88.6)	46 (38.3)	9 (18.8)	
White	76 (28.2)	15 (25.9)	3 (6.8)	28 (23.3)	30 (62.5)	
Other	62 (23.0)	9 (15.5)	2 (4.6)	44 (36.7)	7 (14.6)	
Unknown	7 (2.5)	3 (5.1)	0 (0)	2 (1.7)	2 (4.1)	
Ethnicity						<.001∗
Non-Hispanic or Latino	184 (68.2)	45 (77.6)	43 (97.7)	62 (51.7)	34 (70.8)	
Hispanic or Latino	64 (23.7)	13 (22.4)	1 (2.3)	36 (30.0)	14 (29.2)	
Other	7 (2.6)	0 (0)	0 (0)	7 (5.8)	0 (0)	
Unknown	15 (5.5)	0 (0)	0 (0)	15 (12.5)	0 (0)	
Sex at birth						.721
Male	264 (97.8)	57 (98.3)	42 (95.5)	118 (98.3)	47 (97.9)	
Female	6 (2.2)	1 (1.7)	2 (4.5)	2 (1.7)	1 (2.1)	
Insurance status						<.001∗
Medicaid	84 (31.1)	16 (27.6)	26 (59.1)	15 (12.5)	27 (56.3)	
Medicare	7 (2.6)	1 (1.6)	1 (2.3)	3 (2.5)	2 (4.2)	
Private insurance	83 (30.7)	19 (32.8)	15 (34.1)	34 (28.3)	15 (31.2)	
Uninsured	80 (29.7)	11 (19.0)	2 (4.5)	64 (53.3)	3 (6.2)	
Unknown	16 (5.9)	11 (19.0)	0 (0)	4 (3.4)	1 (2.1)	
Symptoms						
Skin lesions	253 (93.7)	54 (93.1)	38 (86.4)	114 (95.0)	47 (97.9)	.120
Oral lesions	28 (10.4)	10 (17.2)	5 (11.4)	8 (6.7)	5 (10.4)	.190
Rectal lesions	58 (21.5)	10 (17.2)	10 (22.7)	26 (21.7)	12 (25.0)	.798
Fever/flu-like Symptoms	152 (56.3)	37 (63.8)	24 (54.6)	56 (46.7)	35 (72.9)	.010∗
Rectal pain	70 (25.9)	14 (24.1)	18 (40.9)	27 (22.5)	11 (22.9)	.102
ESI						
2	33 (12.2)	8 (13.8)	6 (13.6)	16 (13.3)	3 (6.3)	<.001∗
3	153 (56.7)	36 (62.1)	26 (59.1)	58 (48.3)	33 (68.8)	
4	46 (17.0)	10 (17.2)	11 (25.0)	23 (19.2)	2 (4.1)	
5	6 (2.2)	4 (6.9)	1 (2.3)	1 (0.9)	0 (0)	
Unknown	32 (11.9)	0 (0)	0 (0)	22 (18.3)	10 (20.8)	
Suspected transmission						
MSM	146 (54.1)	47 (81.0)	30 (68.2)	39 (32.5)	30 (62.5)	<.001∗
Multiple partners	16 (5.9)	8 (13.8)	3 (6.8)	5 (4.2)	0 (0)	.017∗
Regular partner/spouse	22 (8.2)	13 (22.4)	4 (9.1)	4 (3.3)	1 (2.1)	<.001∗
Causal partner	45 (16.7)	17 (29.2)	3 (6.8)	15 (12.5)	10 (20.8)	.008∗
Close/household contact	13 (4.8)	4 (6.9)	4 (9.1)	3 (2.5)	2 (4.2)	.290
Unknown	95 (35.2)	4 (6.9)	12 (27.3)	65 (54.2)	14 (29.2)	<.001∗

ESI, emergency severity index.

∗ *P* values indicate statistical significance (*P* < .05).

### 3.2 Clinical Characteristics

Patients most commonly presented with skin lesions (253, 94%), fever or flu-like symptoms (152, 56%), and rectal pain (70, 26%; Table 1). The most frequently reported transmission route was “sexual contact—men who have sex with men” (146, 54%), whereas “known exposure to mpox” was less common (30, 11%). Most patients were discharged from the ED (210, 78%), with nearly half referred for primary care follow-up (122, 45%) and about one-third to an infectious disease clinic (79, 29%). The χ^2^ test of independence revealed significant regional differences in fever or flu-like symptoms (χ^2^(3) = 11.29, *P* = .010), with a higher prevalence in the northeast and a lower prevalence in the south. However, rectal pain, skin lesions (χ^2^(3) = 5.84, *P* = .120), oral lesions (χ^2^(3) = 4.76, *P* = .190), and rectal lesions (χ^2^(3) = 1.01, *P* = .798) did not significantly vary by region. MSM contact was significantly more common in the northeast (χ^2^(3) = 44.37, *P* < .001) and less common in the south. Engagement with ≥3 partners without protection was more frequent in the East (χ^2^(3) = 10.19, *P* = .017), whereas the west reported no cases. Sexual contact with a regular partner was also more common in the East (χ^2^(3) = 21.90, *P* < .001), whereas the west had fewer reports. Casual/anonymous contact was significantly higher in the East (χ^2^(3) = 11.85, *P* = .008), whereas the midwest had fewer such reports. Nonsexual contact was infrequent and not significantly different across regions (χ^2^(3) = 3.75, *P* = .290), but other/not sure/unknown contact was significantly higher in the south and west (χ^2^(3) = 41.28, *P* < .001). ESI also varied significantly (χ^2^(12) = 38.81, *P* < .001), with higher ESI (level 2) in the south and midwest, and a higher proportion of nonurgent (level 5) cases in the northeast. Unknown ESI levels were more prevalent in the South and West.

### 3.3 HIV Status and Testing for Coinfections

More than half (141/270, 52%) of individuals diagnosed with mpox had a known diagnosis of HIV when they presented to the ED (Table 2). Of those who were tested for HIV during their ED encounter (n = 65), 15% (10/65) were newly diagnosed at the time of mpox diagnosis, and 85% (55/65) had a negative HIV test. Overall, 10% (28/270) of the cohort had no HIV testing done as part of their ED encounter. Testing for other STIs among patients with mpox varied. Overall, 45% (122/270) were tested for chlamydia, 47% (128/270) for gonorrhea, and 53% (143/270) for syphilis. Of those tested for STI coinfection, 36% (51/143) had positive syphilis serology (not all diagnoses could be confirmed by clinical history), followed by 7% (9/128) gonorrhea and 7% (8/122) chlamydia positivity.

**Table 2:** HIV status and coinfection testing by site.

	Total (N=270)	Johns Hopkins (N=47)	Boston Medical Center (N=11)	University of Chicago (N=26)	University Hospitals (N=18)	UC Davis (N=9)	University of Washington (N=39)	University of Texas (N=98)	University of South Florida (N=22)
HIV Status									
Positive—Previously Diagnosed	141 (52.2)	17 (36.2)	3 (27.2)	19 (73.1)	4 (22.2)	7 (77.8)	23 (59.0)	57 (58.2)	11 (50.0)
Positive—Newly Diagnosed	10 (3.7)	1 (2.1)	0 (0.0)	0 (0.0)	1 (5.6)	0 (0.0)	1 (2.6)	3 (3.0)	4 (18.2)
Negative	91 (33.7)	21 (44.7)	7 (63.6)	7 (26.9)	13 (72.2)	1 (11.1)	13 (33.3)	23 (23.5)	6 (27.3)
Unknown	28 (10.4)	8 (17.0)	1 (9.2)	0 (0.0)	0 (0.0)	1 (11.1)	2 (5.1)	15 (15.3)	1 (4.5)
STI Testing Performed									
HIV (not previously diagnosed)	65 (50.4)	15 (50.0)	3 (37.5)	5 (71.4)	9 (64.3)	1 (50.0)	10 (62.5)	12 (29.3)	10 (91.0)
Chlamydia	122 (45.2)	30 (63.8)	5 (45.5)	20 (76.9)	8 (44.4)	4 (44.4)	20 (51.3)	19 (19.4)	16 (72.7)
Gonorrhea	128 (47.4)	29 (61.7)	5 (45.5)	19 (73.1)	8 (44.4)	5 (55.6)	20 (51.3)	26 (26.5)	16 (72.7)
Syphilis	143 (53.0)	28 (59.6)	6 (54.5)	21 (80.8)	12 (66.7)	4 (44.4)	25 (64.1)	32 (32.7)	15 (68.2)
STI—Concurrent Infections									
HIV (new diagnoses)	10 (15.4)	1 (6.7)	0 (0.0)	0 (0.0)	1 (11.1)	0 (0.0)	1 (10.0)	3 (25.0)	4 (40.0)
Chlamydia	8 (6.6)	1 (3.3)	0 (0.0)	2 (10.0)	1 (12.5)	0 (0.0)	1 (5.0)	1 (5.3%)	2 (12.5)
Gonorrhea	9 (7.0)	1 (3.5)	1 (20.0)	2 (10.5)	2 (25.0)	0 (0.0)	2 (10.0)	1 (3.8)	0 (0.0)
Syphilis	51 (35.7)	10 (35.7)	4 (66.7)	7 (33.3)	9 (75.0)	2 (50.0)	1 (4.0)	14 (43.8)	4 (26/7)

There were major regional site variations noted in rates of HIV and STI cotesting and positivity. HIV testing (excluding those previously diagnosed) among sites varied from 29% to 91%, with HIV positivity rates as high as 40% at 1 site in the south. For chlamydia, the percentage of patients tested ranged from 19% to 77%, and positivity rates varied from 0% to 13%. The percentage tested for gonorrhea ranged from 27% to 73%, with positivity rates from 0% to 25%. Finally, the percentage for syphilis testing ranged from 33% to 81%, with positivity rates from 4% to 75%.

### 3.4 ED Disposition

Overall, 20% (54/270) of patients were admitted, with higher rates among those with greater illness severity and HIV coinfection (Table 3). Logistic regression showed that patients triaged as ESI level 2 had significantly higher odds of admission compared with lower ESI levels (OR: 5.54, 95% CI: 1.66-18.47, *P* = .005), whereas ESI level 3 was not a significant predictor (OR: 1.62, 95% CI: 0.65-4.06, *P* = .301). Patients with HIV coinfection had a higher admission rate (30%) compared with those without HIV (8%), with HIV being a strong predictor of admission (OR: 5.85, 95% CI: 2.28-15.01, *P* < .001). Among symptomatic patients, rectal lesions significantly increased the odds of admission (OR: 3.90, 95% CI: 1.19-12.79, *P* = .025). Age 30-49 years showed higher odds compared with those under 30 years (OR: 2.33, 95% CI: 0.87-6.25, *P* = .091), although not statistically significant. Admission rates did not differ by sex or race. Among admitted patients, 98% (n = 53) had a skin rash, and 67% (n = 36) had fever or flu-like symptoms.

**Table 3:** Logistic regression analysis of factors associated with admission.

	Odds ratio	95% CI	*P* value
Age category (ref: 18-29 y)			
30-49 y	2.33	0.87 - 6.25	.091
50-64 y	3.44	0.72-16.45	.122
Race or ethnicity (ref: White)			
Black or African American	1.05	0.38-2.88	.921
Other	1.38	0.43-4.38	.587
Unknown	0.58	0.04-4.38	.698
Ethnicity (ref: non-Hispanic)			
Hispanic or Latino	0.68	0.21-2.19	.522
Other	3.84	0.47-31.76	.211
Unknown	2.45	0.48-12.52	.280
Sex at birth (Ref: Male)			
Female	1.04	0.06-19.04	.979
Insurance status (ref: private insurance)			
Medicaid	1.58	0.61-4.09	.345
Medicare	6.24	0.69-56.15	.102
Uninsured or unknown	0.68	0.24-1.91	.466
Symptoms			
Skin lesions	0.55	0.10-3.06	.492
Oral lesions	1.86	0.61-5.62	.274
Rectal lesions	3.90	1.19-12.79	.025∗
Fever/flu-like symptoms	0.83	0.39-1.78	.630
Rectal pain	0.33	0.10-1.14	.079
ESI (ref: level 4, 5, or unknown)			
2	5.54	1.66-18.47	.005∗
3	1.62	0.65-4.06	.301
Suspected transmission			
MSM	1.10	0.28-4.25	.896
Multiple partners	0.42	0.06-2.80	.370
Regular partner/spouse	4.25	0.95 -18.92	.058
Causal partner	0.96	0.28-3.27	.942
Close/household contact	1.40	0.23-8.71	.718
Unknown	2.04	0.44-9.49	.366
HIV Status (Ref: Negative)			
Positive	5.85	2.28-15.01	.000∗

ESI, emergency severity index.

∗ P *values indicate statistical significance (*p *< .05)*.

## 4 Limitations

A significant limitation is that the study sites were selected based on convenience sampling and represent only a very small number of EDs across the United States; furthermore, they were only academic institutions. Although our sites did include a broad geographic representation of most regions in the United States, our findings are not fully generalizable to all US EDs. In addition, regions of the United States that are known to have been particularly hard hit with mpox, such as New York City, are not represented here. Another limitation of the study is that some patients with mpox may have been missed if diagnostic testing was conducted at an outside lab, or if laboratory testing was indeterminate. At the start of the mpox outbreak, many EDs had to send patient samples outside of their institution for testing, and many patients did not receive their results until days later. Additionally, result-reporting pathways developed over the outbreak, and some positive tests identified early on may not have been incorporated into the electronic medical record. Finally, because this study was a retrospective chart review, there were likely inconsistencies in data collection techniques when screening patients and collecting demographic information.

## 5 Discussion

In this national multisite retrospective chart review of 270 mpox cases from academic EDs across the United States, we characterized clinical features, demographics, and management strategies of patients presenting to acute care settings. Unlike prior single-site ED studies with small case numbers, our multisite approach allowed for a broader analysis of mpox presentation.^3^^,^^5^^,^^7^, ^8^, ^9^, ^10^, ^11^ Most patients were male and younger. Among those who knew their HIV status, 52% had coexisting HIV infection, exceeding the CDC’s national estimate of 41%.^18^ High rates of syphilis coinfection (36%) and gonorrhea/chlamydia (7%) were also observed.^18^ ED patients with mpox were racially and ethnically diverse, with over half identifying as Black or African American. This contrasts with CDC estimates, where 41% of cases were among non-Hispanic White persons and 26% among non-Hispanic Black persons.^18^ This difference may reflect the demographic profile of ED populations and participating sites or indicate that non-White patients were more likely to be diagnosed in EDs compared with other settings.

The high rates of HIV and mpox coinfection observed in our study underscore the need for concomitant HIV testing in patients with suspected or confirmed mpox in the ED. Notably, 15% of mpox patients had a new diagnosis, far exceeding the typical 0.4% positivity rate for routine HIV screening in health care settings.^19^ Patients with HIV coinfection face worse outcomes and higher mortality with mpox, as evidenced by CDC data indicating that 94% of mpox-related deaths occurred in immunocompromised individuals with HIV.^20^ These findings support a syndemic approach to managing HIV, STIs, and mpox.

Based on our data, we recommend that ED clinicians maintain a high suspicion for HIV coinfection when diagnosing mpox and perform routine HIV and STI screening. Consistent with our findings, the CDC reports that 41% of mpox cases had an STI diagnosis within the preceding year, emphasizing the importance of rapid linkage to HIV care and prevention.^20^

Several of the study sites have implemented screening algorithms for HIV, HCV, and syphilis, using targeted or universal approaches.^21^, ^22^, ^23^, ^24^, ^25^ Effective strategies include chief complaint-based order sets and risk-triggered orders. These approaches can enhance coinfection detection and better prepare EDs for future outbreaks.

The mpox epidemic re-emphasized lessons learned from the COVID-19 pandemic, in particular, the importance of robust and open communication pathways between EDs with state and local public health departments and federal public health agencies. The ED provides critical health information to health departments (often serving as the “canary in the coalmine” and first or early site for identifying an emerging outbreak), particularly for patients belonging to marginalized groups that may have less access to medical care and that may be at a higher risk of infection. Emergency physicians must therefore be prepared to manage similarly novel cases using information provided by their hospital’s division of Infection Prevention and Control, their state and local health departments, and federal organizations such as the CDC’s Health Alert Network. Similarly, public health departments have the responsibility of providing clear concise pathways for the screening, isolation, testing, and follow-up for EDs for those who are not admitted to the hospital, and communication pathways should be well established. Unfortunately, given constraints on resources in both local public health departments and EDs, significant resource challenges remain.

It is not possible to know from the data collected why patients presented to the ED as opposed to other sites for possible diagnosis. ED patients may reflect individuals who do not have access to primary care or infectious disease clinics, or those with acute pain or complications that could not be managed in the outpatient setting. Given the stigma and fear around mpox, as well as the rapid escalation in cases, many patients sought simply to get confirmation of diagnosis and guidance on quarantine and treatment options.

This multisite study highlights key demographic and clinical characteristics of ED patients with mpox, notably the high rates of HIV and/or syphilis coinfection. We recommend that ED clinicians routinely test for HIV and STIs in patients with suspected or confirmed mpox. The high prevalence of uninsured and underinsured patients also underscores the need for robust postdischarge support. By integrating data from multiple sites, this study identifies critical trends for managing mpox and emphasizes the importance of multisite surveillance networks to enhance outbreak response and preparedness.

## Author Contributions

GD, Y-HH, and BH conceptualized and designed the study. GD had primary responsibility for database management and data collection from sites. GD and RCD performed data analyses. GD, KF, KAS, JWD, RCD, and BH primarily interpreted results. GD, KF, and BH primarily drafted the manuscript. All authors contributed substantially to its revision. GD and KF take responsibility for the paper as a whole.

## Funding and Support

By *JACEP Open* policy, all authors are required to disclose any and all commercial, financial, and other relationships in any way related to the subject of this article as per ICMJE conflict of interest guidelines (see www.icmje.org). The authors have stated that no such relationships exist.

## Conflict of Interest

KAS: grants that support her time including K23AI166277 from NIAID, an EMF Health Disparities Grant, and a HRSA or Chicago Department of Public Health grant. JWW: grants supporting his time including Gilead, PCORI, NIDA, and HRSA. Payments for lectures from Astra Zeneca and Janssen, and travel expenses from Gilead. KF: grants supporting time from Gilead. EMSP: grants supporting time from Gilead.
